# Calcium Dynamics of Cortical Astrocytic Networks In Vivo

**DOI:** 10.1371/journal.pbio.0020096

**Published:** 2004-04-13

**Authors:** Hajime Hirase, Lifen Qian, Peter Barthó, György Buzsáki

**Affiliations:** **1**Center for Molecular and Behavioral Neuroscience, Rutgers UniversityNewark, New JerseyUnited States of America

## Abstract

Large and long-lasting cytosolic calcium surges in astrocytes have been described in cultured cells and acute slice preparations. The mechanisms that give rise to these calcium events have been extensively studied in vitro. However, their existence and functions in the intact brain are unknown. We have topically applied Fluo-4 AM on the cerebral cortex of anesthetized rats, and imaged cytosolic calcium fluctuation in astrocyte populations of superficial cortical layers in vivo, using two-photon laser scanning microscopy. Spontaneous [Ca^2+^]_i_ events in individual astrocytes were similar to those observed in vitro. Coordination of [Ca^2+^]_i_ events among astrocytes was indicated by the broad cross-correlograms. Increased neuronal discharge was associated with increased astrocytic [Ca^2+^]_i_ activity in individual cells and a robust coordination of [Ca^2+^]_i_ signals in neighboring astrocytes. These findings indicate potential neuron–glia communication in the intact brain.

## Abstract 2

Two-photon laser scanning microscopy was used to image calcium concentration changes in astrocytes in the cerebral cortex of anesthetized rats

## Introduction

Astrocytes are nonneuronal cells of the brain with some known and hypothesized functions (Kettenmann and Ransom 1995; Fields and Stevens-Graham 2002). Traditionally, astrocytes have been considered to mediate supportive and protective functions in the central nervous system because of their strategic placement relative to the vasculature, and because they lack fast sodium action potentials. It is only recently that this family of glial cells has been implicated in controlling the dynamics of the neuronal networks in the central nervous system (Nedergaard 1994; Parpura et al. 1994; Kang et al. 1998; Parri et al. 2001). Although the membrane potential of unidentified glial cells shows correlated changes with neuronal activity in vivo (Amzica and Steriade 2000; Amzica and Massimini 2002), most of our knowledge on neuron–glia and glia–glia communication comes from studies in vitro.

In cultured and acutely prepared astrocytes, free calcium concentration ([Ca^2+^]_i_) in the cytosol undergoes large changes spontaneously or in response to various physiological and pharmacological manipulations, such as mechanical stimulation, membrane potential depolarization, and activation of metabotropic glutamate receptors (Cornell-Bell et al. 1990a; Pasti et al. 1997). These slow events are mediated by release of Ca^2+^ from intracellular stores (Charles et al. 1993; Venance et al. 1997). The [Ca^2+^]_i_ surges can be evoked by strong neuronal activity (Dani et al. 1992; Porter and McCarthy 1996), suggesting a potential homeostatic role of astrocytes in the regulation of extracellularly accumulating neurotransmitters (Verkhratsky et al. 1998). Conversely, spontaneous [Ca^2+^]_i_ changes in astrocytes have been shown to influence neuronal excitability (Parpura et al. 1994; Kang et al. 1998; Pasti et al. 2001). The mechanism of activity propagation among astrocytes is controversial. In tissue cultures, [Ca^2+^]_i_ events can propagate among a network of astrocytes via gap junction or by elevation of adenosine triphosphate level (Cornell-Bell et al. 1990b; Charles et al. 1991; Nedergaard 1994; Reetz et al. 1997; Newman 2001). In the in vitro slice preparation, coordination of [Ca^2+^]_i_ activity appears independent of gap junctions but may require transmitter activation of N-methyl-D-aspartic acid (NMDA) and/or metabotropic glutamate receptors (Parri et al. 2001; Aguado et al. 2002; Nett et al.2002; Tashiro et al. 2002). Moreover, the extent and magnitude of these network effects vary as a function of the preparation used, and can involve correlated [Ca^2+^]_i_ changes in no, or only a few, neighboring astrocytes, or the whole population (Porter and McCarthy 1996; Verkhratsky et al. 1998). Whether and how the observations in the various in vitro situations apply to the intact brain have yet to be determined.

We have used two-photon laser scanning microscopy (2-PLSM) to monitor cytosolic Ca^2+^ concentration in astrocytes labeled with Fluo-4 acetoxymethyl (AM) ester in juvenile rats in vivo. We find that [Ca^2+^]_i_ dynamics in astrocytes is rather quiescent during baseline anesthesia. However, increased population bursting, brought about by attenuating γ-aminobutyric acid (GABA_A_) receptor-mediated neurotransmission, leads to increased magnitude [Ca^2+^]_i_ surges, and the [Ca^2+^]_i_ changes become more strongly coordinated in neighboring astrocytes.

## Results

### Loading of Calcium-Sensitive Dye

**Figure 1:**
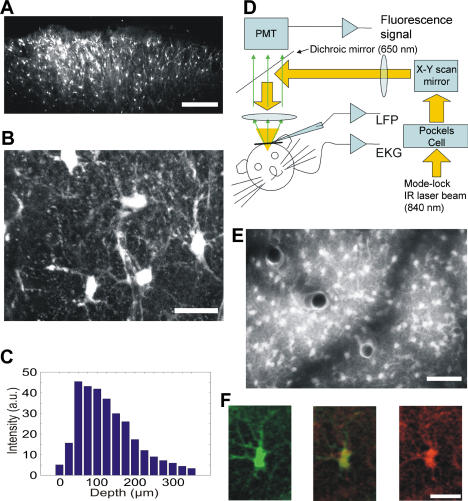
In Vivo Loading and Imaging of Astrocytes Using Fluo-4 AM (A) Acute slice prepared 1 h after dye loading. Scale bar, 200 μm. (B) Higher magnification reveals cells with typical astrocyte morphology. Scale bar, 20 μm. (C) Average bulk fluorescence as a function of the depth from the pial surface. (D) Schematic drawing of the experimental arrangement. Abbreviations: EKG, electrocardiogram. PMT, photomultiplier. LFP, glass micropipe for local field potential and multiple unit recording. The same pipette was used to deliver bicuculline. (E) Image taken 50–150 μm below pial surface in vivo. Flattened xyz stack. (F) Fluo-4 AM loaded cells (left) were stained for S100B immunoreactivity (right), and the images were merged (center). See Video S3 for large-scale staining. Scale bar, 20 μm.

**Figure 2:**
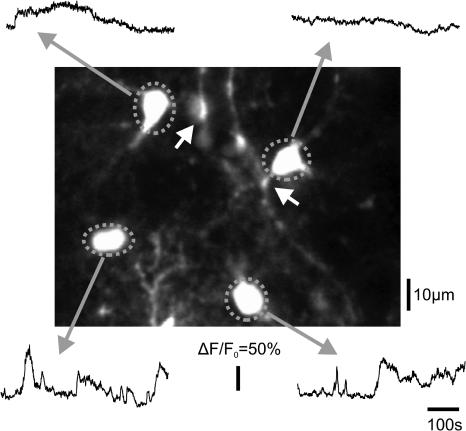
Time-Lapse Imaging of Astrocytes In Vivo Four astrocytes, from which fluorometric Ca^2+^ imaging (0.5 Hz) was made, are outlined. A blood vessel, outlined by the astrocyte end feet, runs diagonally across the viewed area. White arrows show the end foot connected to the imaged astrocyte.

To examine the depth of penetration of the Fluo-4 AM, coronal brain slices (300 μm thick) were acutely prepared after the residual dye was washed off from the craniotomy. A large number of cells below the craniotomy showed fluorescence labeling (Figure 1). On the basis of morphological appearance (see also Videos S1-S4), most brightly labeled cells were astrocytes, in accordance with recent observations using a pressure application of the indicator (Stosiek et al. 2003). The large overlap between Fluo-4 AM-loaded cells and astrocytes identified by S100B immunoreactivity provided confidence that most of the loaded cells were astrocytes (Video S5). In addition to astrocytes, capillary endothelial cells and pericytes, outlining microvessels, were also observed, albeit less regularly. Some processes of astrocytes contacted local vessels. To quantify the dye penetration, mean bulk fluorescence intensity was plotted for different depths from the pial surface. Most intensive labeling occurred between 50–150 μm below the surface (i.e., layers I/II), but labeled cells could be visualized at greater than 300 μm as well (Figure 1C). The decreased fluorescence on the surface is likely due to the diluting effect of the washout procedure in the superficial tissue. Like the histological appearance, in vivo imaging revealed numerous astrocytes (Figure 1E). Although the labeling was dense, the somata and several associated processes, including vessel-contacting end feet, of single astrocytes could be clearly revealed (Figure 2).

### Spontaneous Calcium Events in Astrocytes

In our initial experiments, we made a large number of line scans (sampling rate ∼200 Hz) of dye-loaded cells to examine whether some of them were neurons. We never observed short-lasting [Ca^2+^]_i_ transients (less than 200 ms; Svoboda et al. 1997; Garaschuk et al. 2000), suggesting that the brightly loaded cells were likely to be non-neuronal (Parri et al. 2001; Stosiek et al. 2003). In subsequent experiments (*n* = 8 rats), cells with astrocytic appearance (*n* = 185) were selected for long-term (10–20 min) monitoring. For quantitative studies, three states of [Ca^2+^]_i_ activity were distinguished: (a) quiescent state with very slow (less than 0.025 Hz) oscillations of baseline fluorescence level, (b) [Ca^2+^]_i_ spikes (greater than or equal to 20% increase in *ΔF/F_0_* between 5–50 s), and (c) [Ca^2+^]_i_ plateau potentials (greater than or equal to 20% increase in *ΔF/F_0_* for greater than 50 s). [Ca^2+^]_i_ spikes and [Ca^2+^]_i_ plateau potentials were automatically detected. In the control (baseline) condition, 11% of astrocytes had at least one spike event, and 52% had at least one plateau event in 10 min. The mean frequency of [Ca^2+^]_i_ spikes among the cells that had at least one [Ca^2+^]_i_ spike was 0.121 ± 0.098 per minute (mean width at greater than or equal to 20% *ΔF/F_0_*: 25.1 ± 10.31 s) and the mean frequency of [Ca^2+^]_i_ plateau was 0.118 ± 0.058 per minute (mean duration: 160.4 ± 114.9 s).

To investigate whether the baseline values of [Ca^2+^]_i_ dynamics were affected by increasing neuronal activity, we induced regularly occurring population bursts by local application of bicuculline (Schwartz and Bonhoeffer 2001; *n* = 7 rats). Large amplitude (0.69 ± 0.26 mV) synchronous field events (approximately 100 ms) occurred at relatively regular frequency (0.15 ± 0.06 Hz), associated with multiple unit discharges. No significant difference was observed in average heartbeat frequency between the control sessions and bicuculline sessions (4.51 ± 0.54 Hz and 4.36 ± 0.74 Hz, respectively; paired t-test, *p* = 0.13).

We used two methods to evaluate the effect of neuronal activity on [Ca^2+^]_i_ in astrocytes (*n* = 214 cells). First, the incidence of [Ca^2+^]_i_ spikes and plateau events was counted in the absence and presence of bicuculline-induced population bursts. Under bicuculline condition significantly more astrocytes had [Ca^2+^]_i_ spikes (11% versus 24%; *p* < 0.001; Fisher's exact test), whereas the probability (52% versus 54%) of plateau potentials did not differ significantly. The mean duration of plateau potentials, however, was significantly longer (160.4 ± 114.9 s versus 211.12 ± 152.175 s; t-test, *p* < 0.001) after bicuculline treatment. Among the cells that exhibited at least one spike or plateau event, there was not a significant difference in frequency of the event occurrences (spike: 0.121 ± 0.098/min versus 0.098 ± 0.068/min; t-test, *p* = 0.24; plateau 0.118 ± 0.058/min versus 0.112 ± 0.049/min; t-test, *p* = 0.46). Thus, the major difference between control and bicuculline conditions was the higher proportion of active astrocytes under bicuculline.

**Figure 3:**
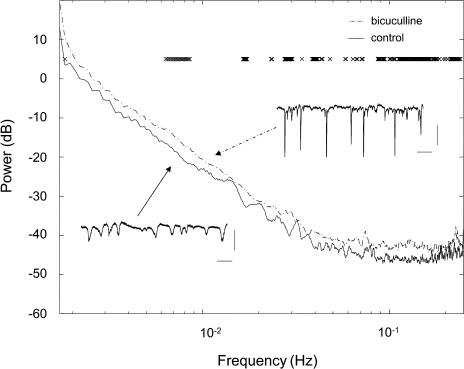
Frequency Domain Analysis of Population Dynamics of Fluorescence in Astrocytes in Control State and during Bicuculline-Induced Neuronal Hyperactivity Insets show local field potentials in a control animal and regular spiking in a bicuculline treated mouse (scale bar: 2.0 s, 500 μV). Asterisks show significant differences (*p* < 0.05) between groups at various frequencies.

The second method examined [Ca^2+^]_i_ changes in the frequency domain. The *ΔF/F_0_* trace was considered as a continuous process, and the power spectrum estimate was calculated with a multi-taper method for each astrocyte and averaged across cells. There was a general increase of power at all frequencies in bicuculline-treated animal. The most consistent significant increase (*p* < 0.05) of power appeared in the frequency range of 0.10–0.24Hz, reflecting the increased incidence of [Ca^2+^]_i_ spikes. Short-term cross-correlation of neuronal field bursts and [Ca^2+^]_i_ signals (± 10 s) did not show a significant time-locked relationship (Figure 3).

### Spatio-Temporal Dynamics of [Ca^2+^]_i_ Events

**Figure 4:**
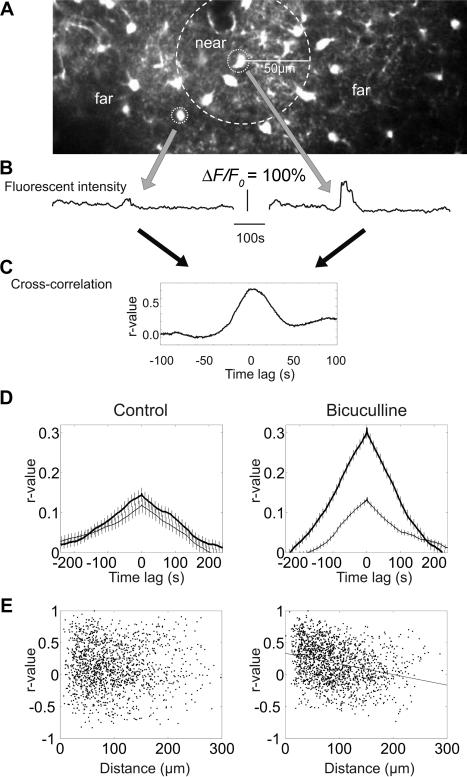
Spatio-Temporal Dynamics of Astrocyte Ca^2+^ Activity (A) Definition of nearby (less than 50 μm) and distant (greater than 50 μm) cell pairs. (B) Fluorescence changes in two nearby astrocytes. (C) Cross-correlogram of fluorescent intensity. (D) Mean cross-correlation of *ΔF/F_0_* in all nearby (thick line) and distant (thin line) cell pairs in control condition (left) and in the presence of bicuculline (right). Note large increase of *ΔF/F_0_* correlation in nearby cell pairs in the bicuculline condition (error bar: standard error of the mean). (E) Relationship between distance of the two cells and the magnitude of correlation at zero timelag. Note lack of a reliable relationship in the control condition (left). Note also the significant negative correlation between the distance and correlated *ΔF/F_0_* changes in cell pairs in the bicuculline-treated cortex (right).

In individual experiments, propagation of synchronous activity could be observed visually (Figure 4A; Video S6) but the spatio-temporal relationship of [Ca^2+^]_i_ dynamics among astrocytes varied across experiments. To quantify the magnitude and spatial extent of this population effect, pair-wise cross-correlograms of *ΔF/F_0_* intensity were calculated separately for nearby cell pairs (local: less than or equal to 50 μm) and distant cell pairs (greater than 50 μm). In control conditions, the temporal correlation of [Ca^2+^]_i_ signals in neighboring pairs was somewhat larger than in distant pairs, but this difference was not significant (*n* = 374 neighbor pairs and *n* = 1,138 distant pairs). Nevertheless, [Ca^2+^]_i_ signals in astrocytes were not completely random, since the cross-correlograms had wide central peaks at the 10–100 s scale (Figure 4B). In contrast to the baseline condition, the temporal correlation of [Ca^2+^]_i_ changes in local and distant pairs were significantly different after large population bursts were brought about by bicuculline (Figure 4C). Correlation of distant pairs under bicuculline (*n* = 433 pairs) was similar to those in the control condition. However, synchrony between local pairs (*n* = 1,282) increased several-fold relative to both distant pairs under the same condition (*t*-test, *p* < 0.0001) and to local pairs in the baseline condition (*t*-test, *p* < 0.0001).

Using a different approach, the magnitude of the zero-timelag correlation coefficient for each cell pair was plotted against distance between the cell pairs. Under control condition, no notable relationship was observed between these variables (Figure 5; *n* = 1,512 cell pairs, *r* = 0.019, *p* = 0.46). In contrast, a significant negative correlation was found between the synchrony of [Ca^2+^]_i_ signals in the bicuculline condition (*n* = 1,715; *r* = −0.281; *p* < 0.0001).

## Discussion

Astrocytes in superficial cortical layers were successfully loaded using Fluo-4 AM by surface application up to 350 μm from the pial surface in juvenile rats. In agreement with previous literature (Parri et al. 2001; Dallwig and Deitmer 2002; Simard et al. 2003), the majority of the Fluo-4-loaded cells exhibited astrocytic morphology with multipolar branching and bushy microprocesses impinging on local vasculature. 2-PLSM imaging revealed spontaneous [Ca^2+^]_i_ events in individual astrocytes in vivo. Some coordination of these events was indicated by the broad cross-correlograms in the baseline condition. Increased neuronal discharge was associated with increased astrocytic activity and a robust coordination of [Ca^2+^]_i_ signals in neighboring astrocytes, providing evidence for neuron–glia communication in the intact brain.

The magnitude, frequency and pattern of [Ca^2+^]_i_ events observed here are qualitatively similar to those described in tissue cultures (Dani et al. 1992; Charles 1998) and acute hippocampal, neocortical, and thalamic slice preparations (Parri et al. 2001; Aguado et al. 2002; Nett et al.2002; Tashiro et al. 2002). It has been reported that the percentage of active astrocytes in brain slices showed a 2- to 3-fold decrease from early postnatal days to juvenile age (Parri et al. 2001; Aguado et al. 2002). In our experiments, a large portion of the imaged astrocytes were active, showing either [Ca^2+^]_i_ or plateau potentials. It is unlikely that the elevated activity in vivo is due to anesthesia because urethane is known to suppress transmitter release from presynaptic vesicles and attenuate both α-amino-3-hydroxy-5-methylisoxazole-4-propionic acid (AMPA) and NMDA receptors (Hara and Harris 2002). Since blockade of these receptors decreases astrocytic [Ca^2+^]_i_ activity in vitro (Parri et al. 2001; Aguado et al. 2002), it is expected that in the drug-free animal the percentage of active cells will be even higher. A different explanation for the lower percentage of active astrocytes in the slice, relative to the in vivo situation and tissue culture preparation, is that the trauma of brain slicing attenuates spontaneous [Ca^2+^]_i_ activity. Reactive astrocytes in a stab wound area show very limited [Ca^2+^]_i_ activity (Aguado et al. 2002). In addition, the temperature at which the cells are kept may be playing an important role.

In the absence of provoking conditions, spontaneous [Ca^2+^]_i_ activity in individual astrocytes does not spread among astrocytes as an intercellular Ca^2+^ wave (Nett et al.2002). In baseline condition, the magnitude of correlated activity in nearby and distant astrocytes was quite similar. Nevertheless, the presence of zero-timelag correlation suggests that activity in the astrocytic syncytium in vivo is not random, but is under some coordinated control. Widespread but limited coordination of glial cells can be brought about by common synchronizing inputs in the intact brain, such as vascular and vegetative nervous system control or large-scale slow changes of neuronal excitability. The latter possibility is supported by the observation that ionotropic glutamate receptor antagonists and tetrodotoxin effectively decorrelated the astrocytic network without altering the number of active astrocytes (Aguado et al. 2002). Furthermore, the intact corticothalamic system displays substantial excitability fluctuation at the time scale of the astrocytic [Ca^2+^]_i_ events (Jando et al. 1995).

Although neuronal activity is not needed to generate [Ca^2+^]_i_ surges in astrocytes (Aguado et al. 2002; Nett et al.2002), neurotransmitters can enhance the frequency of such events. The impact of neuronal activity on the glial network is illustrated by the increased activity and enhanced local correlation of [Ca^2+^]_i_ signal in astrocytes after regular population bursting of neurons was brought about by the GABA_A_-receptor blocker bicuculline. These changes shared similarities to those observed in hippocampal and neocortical slices (Aguado et al. 2002; Tashiro et al. 2002). In contrast to the slice situation, we did not find a time-locked triggering of astrocytic events to the neuronal bursts (see also Nett et al.2002). This discrepancy may be explained by the magnitude of the evoked neuronal bursts. Bicuculline in vitro evoked rare (greater than 30 s intervals), but very large bursts or afterdischarges (Tashiro et al. 2002; Aguado et al. 2002). In vivo, synchronous events of moderate size occurred frequently (approximately 0.3 Hz). The enhanced bursts, associated with large field potentials, can be regarded as interictal epileptic spikes (Schwartz and Bonhoeffer 2001), but seizures were never observed. Although the exact mechanisms of neuron–astrocyte signaling remain to be disclosed, our findings indicate that neuronal and glial networks are coupled in the intact brain.

Many of the imaged astrocytes had processes (end feet) in close contact with small brain vessels (Peters et al. 1970). It has been shown that surges of [Ca^2+^]_i_ in astrocytes trigger the release of vasoactive compounds (Bezzi et al. 1998). Furthermore, stimulation of single astrocytes in cortical slices led to delayed (greater than 30 s) and protracted dilation of the contacted arteriole (Zonta et al. 2003). These findings support the view that a cardinal function of astrocytes in the intact brain is to regulate local circulation according to the metabolic needs of neurons. Overall, the approach introduced in this paper will be a potent tool to investigate these issues in vivo.

## Materials and Methods

### 

#### Subjects and surgery

Male and female rats, 12–16 d postnatal (P12– P16), of the Sprague–Dawley strain were used in these experiments. Animals were deeply anesthetized with 1.7 g/kg urethane. An outline of the craniotomy above the primary somatosensory (barrel) cortex was marked with a dental drill. A metal frame, similar to what has been described in Kleinfeld and Denk (2000), was attached to the skull with cyanoacrylic. A craniotomy (3–4 mm diameter), centered at 1.5 mm posterior to bregma and 2.5 mm from midline, was performed and the dura mater was surgically removed. Care was taken to avoid any damage to pial vessels or the cortex.

#### Dye loading

Fluo-4 AM (F-14201, 50 μg; Molecular Probes, Eugene, Oregon, United States) was mixed with 2 μl of Pluronic (P-3000, Molecular Probes) and 5 μl of dymethyl sulfoxide (D-8779; Sigma, St. Louis, Missouri, United States) for 15 min. The solution was then diluted in 18 μl of artificial cerebrospinal fluid (ACSF) (125 mM NaCl, 3 mM KCl, 10 mM glucose, 26 mM NaHCO_3_, 1.1 mM NaH_2_PO_4_, 2 mM CaCl_2_, 1 mM MgSO_4_; pH adjusted to 7.4) and mixed for a further 15 min. A small volume (up to 12 μl) of the dye-containing solution was applied to the cortical surface by a micropipette. The solution was retained in place by a small piece gelfoam. The unbound dye was removed 45–60 min after the surface application of Fluo-4 AM by irrigating the exposed surface with ACSF for at least 10 min. The craniotomy was then covered with 1% agar dissolved in phosphate-buffered saline (pH 7.4), and a glass coverslip was placed on a metal frame. This arrangement allowed access for a glass recording electrode from the side. Juvenile rats (P13–P15) were used because we found in preliminary experiments that in adult animals, mostly vascular cells were loaded with the current protocol.

#### Electrophysiological recording

During the recording session, a heating blanket was placed under the rat to maintain body temperature at approximately 37°C. The electrocardiogram (EKG) was monitored continuously. The R wave of EKG was used to monitor brain pulsation-derived movement of artifacts during imaging. Population bursts of cortical neurons (“interictal” spikes; Schwartz and Bonhoeffer 2001) were induced by inserting a large-tip (20–50 μm tip diameter) glass pipette, containing 2 mM bicuculline in 0.9% (w/v) NaCl, into the deep layers of the somatosensory cortex. This electrode also served to record local field potential and multiple unit activity. Large population bursts were reliably induced 10–30 min after the insertion of the pipette.

#### Imaging

A custom-made 2-PLSM was constructed as described earlier (Majewska et al. 2000). In brief, a Ti:S laser (Mira 800F; Coherent, Santa Clara, California, United States) was pumped by a solid state CW laser (Verdi 8; Coherent) to produce a mode-locked beam (840 nm; approximately 100 fs pulse width at 76 MHz repetition rate). The beam was directed to a modified confocal scanhead (Fluoview 300; Olympus, Tokyo, Japan). The fluorescent signal was first filtered with an emission filter (HQ525, passband 525 ± 25 nm; Chroma, Rockingham, Vermont, United States) and detected by an external photo-multiplier tube (R-3896, Hamamatsu Photonics, Hamamatsu City, Japan) with a built-in preamplifier board (F-5 PSU-B; Olympus).

#### Data analysis

Fluorescence signal was quantified by measuring the mean pixel value of a manually selected somatic area for each frame of the image stack using ImageJ software. The values were exported to MatLab and the fluorescence change *ΔF/F_0_* was computed, where *F_0_* is the mean of the lowest 20% of the somatic fluorescence signals. Sessions that had visible drifts when image sequences were replayed as animation (the majority of the cells showed correlated activity [*|r|* > 0.6], or greater than 10% fluorescence change due to the heartbeat when the cell was imaged in line scan [approximately 200 Hz]) were excluded from the analysis. For display purposes, the signal was convolved with a Hanning window of order three to smooth the signal trace. Power spectra of fluorescent signals were computed using the multi-taper method *(NW = 4)*. For the calcium event detection, *ΔF/F_0_* signal was convolved with a Hanning window of order 15. “Spike” events were defined as transient increase of *ΔF/F_0_* signal exceeding 20%, lasting 5–50 s. “Plateau” events were defined as sustained increase of *ΔF/F_0_* (greater than 20%) signal longer than 50 s. Peak amplitudes of both spike and plateau events required an increase of at least 50% *ΔF/F_0_* from the onset of events. Calcium events were automatically detected with the above detection. Cross-correlation between cell pairs was computed by normalizing the *ΔF/F_0_* signals to unity (zero mean, unity standard deviation) so that the computed values represent the correlation coefficient between the two signals at a given timelag. All numbers are indicated as mean ± standard deviation, unless otherwise noted.

#### Immunocytochemisty

Since Fluo-4 AM loading was best visible in the somatic region of the putative astrocytes, we chose S100B antibody (A5110; DakoCytomation, Glostrup, Denmark) because this antibody stains the somatic region of astrocytes as well as its processes (Ren et al. 1992). Following Fluo-4 AM loading, acute brain slices (300 μm thickness) were cut coronally around the dye-loaded area using standard procedures. Fluo-4 in cells of the acute brain slices were fixed by incubating the acute brain slices in freshly made saline containing 40 mg/ml 1-ethyl-3-(3-dimethylaminopropyl)-carbodiimide hydrochloride (EDAC, E7750; Sigma) for 30 min. Next, the slices were incubated in formalin-based fixative (4% formaldehyde, 0.1 M phosphate buffer, [pH ∼7.1]) for 30 min. Once the fixation procedures were completed, the sections were mounted on a glass slide and imaged with 2-PLSM (z-stack; wavelength, 840 nm). After imaging of calcium-loaded cells, and three subsequent washes in phosphate-buffered saline (PBS) (1.06 mM KH_2_PO_4_, 155.17 mM NaCl, 2.96 mM NaHPO_4_, pH approximately 7.4), the slices were treated with S100B antibody (made in rabbit, 1:50 dilution) in Triton X-PBS (0.5% Triton X in PBS) overnight. The sections were subsequently washed three times in PBS, followed by incubation with the secondary fluorescent antibody (1:1000 dilution, 711-166-152, CY3 Anti-Rabbit IgG [H + L]; Jackson ImmunoResearch Laboratories, West Grove, Pennsylvania, United States) in Triton X-PBS solution for 2 h. Simultaneous viewing of the two image stacks allowed a systematic comparison of the extent of overlap between Fluo-4 loading and S100B immunoreactivity (Video S5).

## Supporting Information

Video S1 Visualization of Loaded Astrocytes (Low Magnification) The primary somatosensory cortex (P15) was stained with Fluo-4 AM in vivo and subsequently imaged in vitro. Acute slices (approximately 300 μm thickness) were cut in cold ACSF after the cells were loaded in vivo. (Z step = 1 μm; scale bar = 50 μm).
(49 MB AVI).

Video S2 Visualization of Loaded Astrocytes (High Magnification, Layer I) Same slice as shown in Videos S1, but with higher magnification. Z step = 1 μm; scale bar = 20 μm.
(48 MB AVI).

Video S3 Visualization of Loaded Astrocytes (High Magnification, Layers II/III) Detailed imaging of in vivo-loaded acute slice preparation of the primary somatosensory cortex (P15; approximately 270 μm below the pial surface). Z step = 1 μm; scale bar = 20 μm.
(39 MB AVI).

Video S4 High-Contrast Image Upper Layers (I to II/III) of the Fluo-4 AM-Loaded Somatosensory Cortex (P15) Empty circles in layers II/III, presumed unloaded neurons (note their absence in layer I). The loaded cells have typical glial morphological appearance. Z step = 1 μm; scale bar = 50 μm.
(50 MB AVI).

Video S5 Double-Labeling of Fluo-4 AM-Loaded Astrocytes with S100B Antibody Acute slices (300 μm thickness) were prepared from the in vivo Fluo-4 AM-loaded somatosensory cortex. The slices were subsequently incubated in EDAC containing saline followed by formalin fixation. The loaded astrocytes were identified by examination at various depths and numbered (left). Next, the slices were processed for immunocytochemistry with astrocyte marker S100B. Depth scans (1 μm between the frames) were taken again to determine immunoreactivity of cells with S100B (right movie). An overlapping set of the cells was identified to be S100B-immunoreactive, indicating that nearly all Fluo-4 AM-loaded cells were astrocytes.
(5 MB AVI).

Video S6 Imaging of Fluo-4 AM Fluorescence Activity in Astrocytes In Vivo Movie taken from a P14 rat. Image was taken with 2 Hz sampling rate for 10 min and compressed to 36 s for display purposes. Note spatial- and light-emission-stability of the recorded cells. Note also that at frames approximately 9 s and 15 s, two of the astrocytes in the middle display transient increased fluorescence. Scale bar 50 micro μ.
(55 MB AVI).

## Abbreviations

AM: acetoxylmethyl
AMPA: α-amino-3-hydroxy-5-methylisoxazole-4-propionic acid
GABA: γ-aminobutyric acid
NMDA: N-methyl-D-aspartic acid
P: days postnatal
2-PLSM: two-photon laser scanning microscope
